# Impact of early administration of LAIs on socioeconomic status of patients with long term schizophrenia

**DOI:** 10.1192/j.eurpsy.2025.2201

**Published:** 2025-08-26

**Authors:** U. M. Battelino, A. Rojs, U. Ogrizek, A. Čelofiga

**Affiliations:** 1 Faculty of Medicine University of Maribor; 2 Maribor University Medical Centre, Maribor, Slovenia

## Abstract

**Introduction:**

Schizophrenia is a chronic psychiatric disorder with a complex and diverse pathology (Owen et al. 2016). Currently, the main form of treatment is antipsychotic therapy (AP), which involves the use of either oral antipsychotics (OAP) or long-acting injectable antipsychotics (LAIs) (Stępnicki et al. 2018). Although it affects only about 1% of the population, schizophrenia represents an important socioeconomic problem. The employment rates among individuals with schizophrenia are notably low, with only 10–30% maintaining employment, while approximately 80% are reliant on disability pensions (Holm et al. 2021).

**Objectives:**

The aim of the study was to analyse the association between early administration of LAIs and the socioeconomic status in patients with long lasting schizophrenia and compare the differences between female and male patients.

**Methods:**

This is a retrospective clinical study. We collected socioeconomic and sociodemographic data on all individuals and reviewed each patient’s medical history from their first hospitalization up to the study period. Then we analysed the data. The inclusion criteria were ages from 18 up to 65 years, the diagnosis of schizophrenia or other psychotics disorders for at least 5 years, use of AP therapy for at least 5 years and current use of LAIs for at least 6 months. Exclusion criteria included the diagnosis of treatment-resistant schizophrenia, or the diagnoses classified under F70–79 in the ICD-10 classification.

**Results:**

From the sample of 100 patients, the ones with early initiation of LAIS, within 2 years of onset of disease, had statistically higher rates of employment than those with late initiation of LAIs (p=0,04). We could not prove that the rate of employment was higher among early initiation female patients (p = 0,825), but we could for the male patients (p = 0,005) (Image 1). Meanwhile we couldn’t prove the same for financial independence (p = 0,398), also there was no statistically significant result based on sex (p = 0,367, p = 0,692).

**Image 1:**

**Figure:**
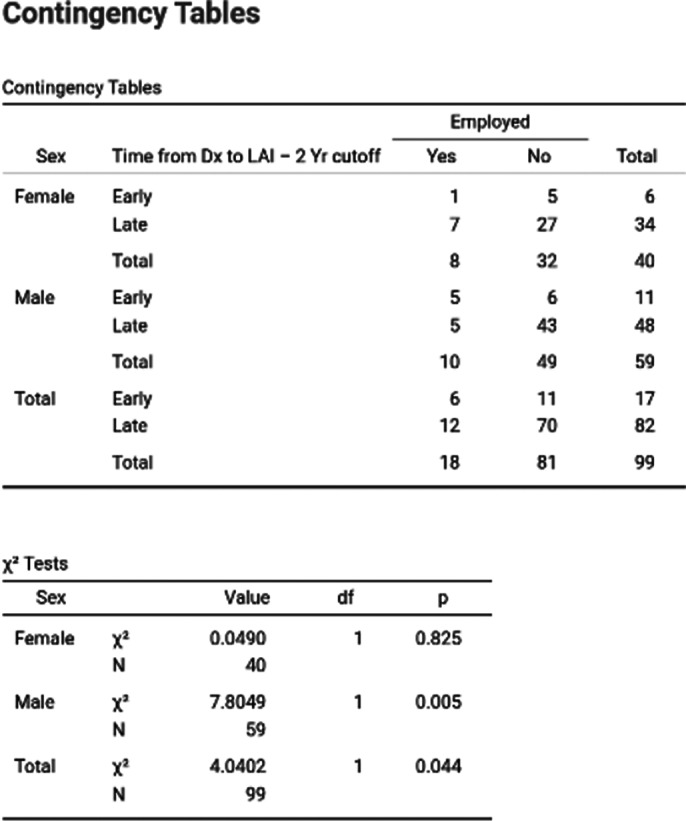

**Image 2:**

**Figure:**
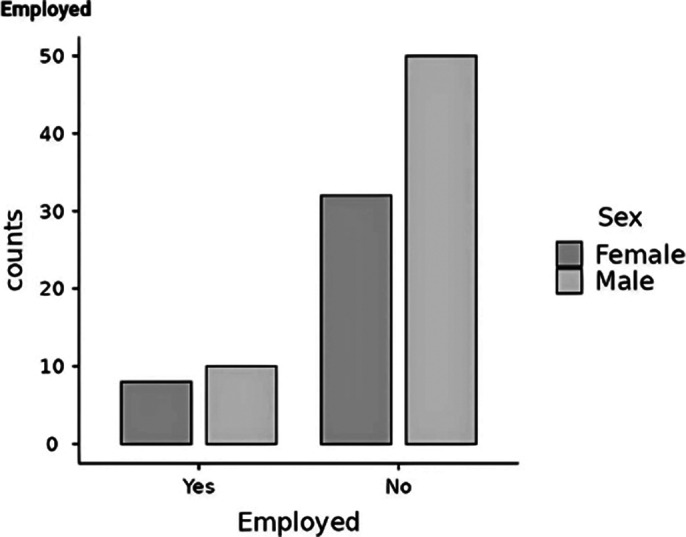

**Image 3:**

**Figure:**
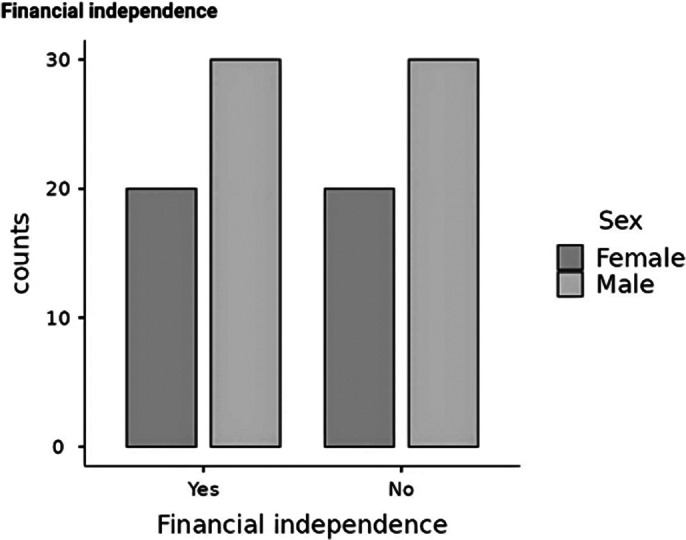

**Conclusions:**

Results suggest that patients with earlier initiation of LAIs have higher rates of employment. The difference between sexes (Image 2 and 3) is also an interesting part of the study as it shows that female patients have a later onset than male patients and therefore receive the first does of LAIs at a much older age than men, which could be influencing the higher rate of unemployment and insufficiency among female patients.

**Disclosure of Interest:**

None Declared

